# Unexpected course of urethroplasty in patient with penile prosthesis

**DOI:** 10.5339/qmj.2026.16

**Published:** 2026-03-22

**Authors:** Ibrahim Alnadhari, Muammer Alshrani, Nabil Moohialdin, Abdelrahman Mohamed, Omar Ali, Osama Abdeljaleel, Ahmad Shamsodini

**Affiliations:** 1 Urology Section, Department of Surgery, Al Wakra Hospital, Hamad Medical Corporation, Al Wakra, Qatar; 2 Department of Surgery, Qatar University, Doha, Qatar *Email: ibrahimah1978@yahoo.com

**Keywords:** Urethra, urethral stricture, recurrence, penile prosthesis

## Abstract

**Introduction:**

Urethral stricture disease is a complex and debilitating condition that significantly impairs urinary function and quality of life. Urethroplasty remains the gold-standard treatment, especially for complex, long-segment, or recurrent strictures. However, performing urethroplasty in patients with penile prostheses presents unique surgical challenges due to the associated anatomical and vascular considerations.

**Case presentation:**

We report the case of a male patient with a history of penile prosthesis implantation nine years earlier who presented with a recurrent bulbar urethral stricture. The patient underwent excision and primary anastomosis (EPA) urethroplasty. Postoperatively, he developed significant urinary leakage at the anastomotic site, which was confirmed by pericatheter urethrogram, and urine culture grew extended-spectrum beta-lactamase (ESBL)-producing Escherichia coli. The condition was managed conservatively with intravenous antibiotics and suprapubic catheter placement, resulting in resolution of the leakage and preservation of prosthesis function.

**Discussion:**

This case highlights the interplay between urethral vascular compromise and the presence of a penile prosthesis, both of which can increase the risk of complications following urethroplasty. Infection, impaired healing, and altered anatomy contribute to the complexity of surgical management. Although the urethral stricture was amenable to EPA, the healing process was likely adversely affected by local infection and compromised blood supply.

**Conclusion:**

Urethroplasty in patients with a penile prosthesis requires a tailored approach that accounts for altered anatomy and vascularity. In this case, the EPA was complicated by infection and urine leakage; however, conservative management resulted in successful healing, restoration of urinary flow, and preservation of prosthesis function. This case highlights the importance of careful surgical planning and flexible postoperative care.

## 1. INTRODUCTION

Urethral stricture disease is a complex and debilitating condition that significantly impairs urinary function and quality of life. Urethroplasty remains the gold-standard treatment for complex, long-segment, or recurrent strictures, providing superior long-term outcomes compared to endoscopic approaches such as dilation or urethrotomy.^1,2^

The normal urethral spongiosum is composed primarily of type I collagen (approximately 75%) and type III collagen (approximately 25%), and an intact neural supply appears essential for maintaining its unique ultrastructure.^3^ Urethral stricture disease represents a fibrotic remodeling process of the subepithelial tissue within the corpus spongiosum, resulting in progressive narrowing of the urethral lumen.^4^ During penile prosthesis implantation, the paired corporal cylinders are connected to a scrotal pump via tubing; unintentional rotation of a cylinder from its neutral axis may twist the tubing, compress the urethra, and induce localized inflammation within the corpus spongiosum, thereby predisposing to urethral stricture formation.^5^ Excision and primary anastomosis (EPA) of the affected urethral segment, with re-approximation of healthy ends, yields long-term success rates exceeding 90%.^6^ When a tension-free anastomosis is not feasible, substitution urethroplasty using penile fasciocutaneous flaps or grafts (e.g., buccal mucosa or genital skin) remains the preferred reconstructive option.^7^ Less invasive treatments, such as direct visual internal urethrotomy (DVIU) or urethral dilation, may be considered; however, they are associated with variable long-term outcomes. Current AUA and EAU guidelines advise against repeated DVIU in patients with long (>2 cm), multiple, penile or distal strictures, or those with extensive spongiofibrosis, due to the low probability of durable success.^2,8^

Urethral vascularity presents a significant concern during urethroplasty, particularly in patients with a penile prosthesis. Erectile dysfunction, the primary indication for prosthesis implantation, is often associated with impaired penile blood supply, and the prosthesis itself may further compromise vascular integrity. By occupying space within the corpora cavernosa, the device can exert pressure on adjacent vascular structures. The urethra receives its blood supply primarily from the dorsal and bulbar arteries, which can be at risk of damage due to scar tissue or compression caused by the implanted device.^5^ These factors can compromise the blood flow to the urethra, potentially affecting the surgical procedure outcomes.

Urethroplasty in patients with penile prostheses presents unique challenges owing to the anatomical proximity of the prosthesis components to the urethra and the increased risk of complications, including infection and prosthesis malfunction.^5,9^ While penile prosthesis implantation and urethroplasty have been well studied as individual procedures, there is a paucity of literature exploring their interplay. Our case report describes a patient who underwent EPA urethroplasty in the presence of a functioning penile prosthesis. We outline the surgical approach, perioperative course, and postoperative complications encountered, emphasizing the importance of tailored planning and flexible management strategies. This study was approved by the Medical Research Center at Hamad Medical Corporation, Qatar (MRC-04-25-094).

## 2. CASE PRESENTATION

A 50-year-old male patient presented to the urology clinic at a public hospital with complaints of a weak urinary stream and a sensation of incomplete bladder emptying. He reported a history of intermittent urethral discharge over a prolonged period. The patient had undergone penile prosthesis implantation (AMS 700 CX) nine years earlier, which was currently functioning well. Two years prior, he was diagnosed with a bulbar urethral stricture and treated with paclitaxel-coated balloon dilatation (Optilum®). His medical history was significant for hypertension.

**Figure 1:**
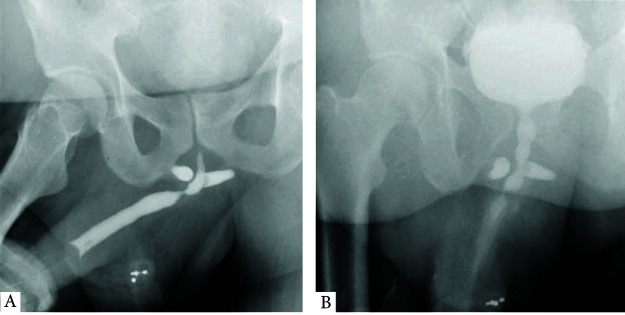
(A) Ascending urethrogram. (B) Micturating urethrogram showing bulbar urethral stricture.

**Figure 2:**
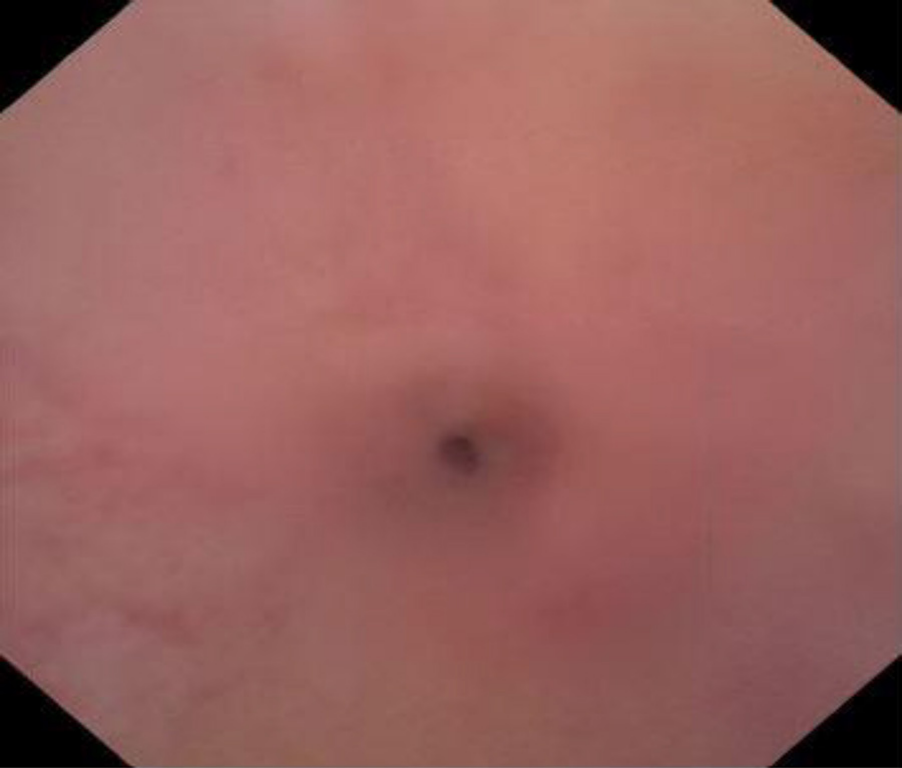
Diagnostic cystoscopy showing severe bulbar urethral stricture.

On physical examination, his abdomen was soft, and the external genitalia appeared normal. Urine flowmetry revealed a box-shaped pattern with a maximum flow rate (Qmax) of 5 ml/s and a post-void residual urine volume of 147 ml. Urine culture showed no growth. An ascending and micturating urethrogram showed a bulbar urethral stricture (Figures 1A and B). Diagnostic cystoscopy showed severe narrowing of the bulbar urethra (Figure 2).

**Figure 3:**
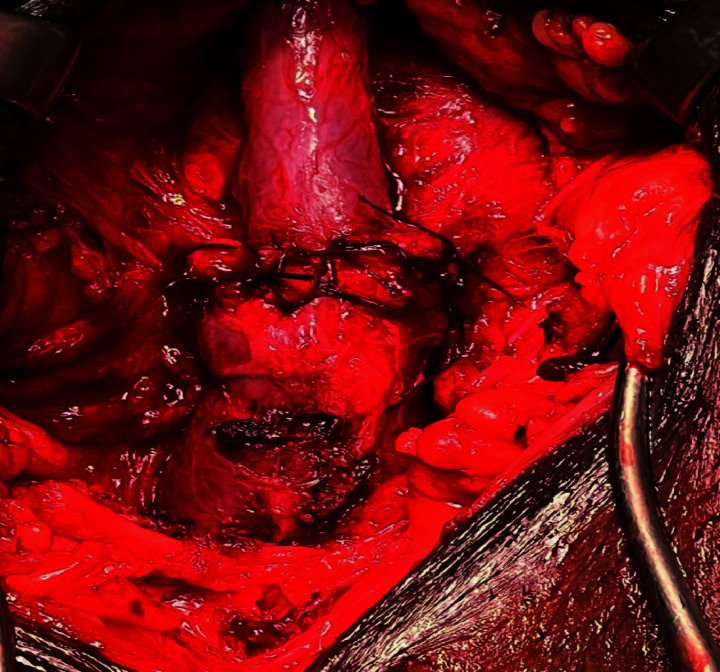
Intraoperative view after excision and primary anastomosis, showing good vasculature of the bulbar urethra.

**Figure 4:**
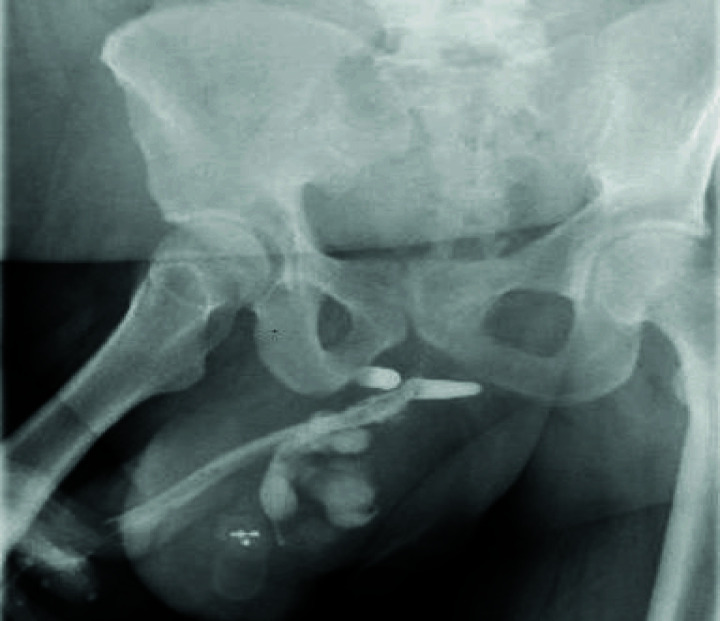
Pericatheter urethrogram, performed two weeks postoperatively, showing significant contrast extravasation at the anastomotic site.

The patient was prepared and electively underwent EPA urethroplasty, which included excision of the stricture segment and end-to-end anastomosis over a 16 Fr silicone catheter (Figure 3). Although good vascularity was noted intraoperatively, the presence of the prosthesis limited urethral mobility. Furthermore, the proximity of the implant to the bulbar urethra raised concerns regarding potential compromise of the local blood supply. A pericatheter urethrogram performed two weeks postoperatively revealed significant extravasation at the anastomotic site (Figure 4). Cultures from the periurethral discharge and urine showed extended-spectrum beta-lactamase (ESBL)-producing Escherichia coli. The patient was treated with intravenous ertapenem 1 g daily for 10 days. The urethral catheter was retained for an additional 10 days and then removed. Following catheter removal, the patient initially passed urine freely.

Two days later, the patient presented with urine leakage from the perineal incision. There was no tenderness or collection in the scrotum or penis. A suprapubic catheter (SPC) was inserted, which successfully controlled the leakage. The SPC was kept in place for four weeks and was then clamped, allowing the patient to pass urine freely. It was subsequently removed. The penile prosthesis remained functional without mechanical failure or infection. The patient was followed in the urology clinic for four months, during which uroflowmetry showed a stable maximum urinary flow rate (Qmax) of 14 ml/s, with no other complications observed.

## 3. DISCUSSION

Urethroplasty in patients with a penile prosthesis presents unique challenges due to the presence of the implant. The case presented highlights the complexity of managing urethral strictures in such patients. The vascularity of the urethra is a critical consideration in such cases. The prosthesis may alter normal anatomy, making surgical access more challenging and potentially affecting the blood supply to the urethra. This can increase the risk of complications such as urethral injury, device erosion, and infection.^4,5^ Ensuring adequate blood supply during urethroplasty is therefore essential to promote healing and reduce the likelihood of postoperative complications.

A simultaneous approach of urethral repair and reimplantation of the penile prosthesis has shown promising results. This method not only addresses the urethral stricture but also ensures the functionality of the prosthesis. Several studies have reported the success of this combined approach. For instance, Cui et al. described a successful single-stage urethroplasty using a penile skin pedicle island flap with reimplantation of a three-piece penile prosthesis.^10^ Similarly, Yi et al. reported the safety and efficacy of synchronous urethroplasty performed during prosthetic surgery.^11^ These findings highlight the importance of a comprehensive surgical plan that addresses both the urethral pathology and the prosthesis management.

Cavalcanti et al. highlighted the difficulties associated with penile prosthesis implantation following urethral stricture reconstruction.^12^ They discussed the potential complications and technical challenges faced by some patients. The authors noted that damage to penile and urethral vascularization, along with corporal fibrosis, can result either from the underlying cause of the stricture or from the urethral reconstructive procedure, thereby contributing to these complications.^12^

The choice of the appropriate urethral reconstructive surgery for bulbar urethral strictures should be individualized, considering factors such as stricture length, location, etiology, prior interventions, and patient comorbidities.^2,8^ In our case, the stricture was short and single; therefore, the decision was to proceed with EPA, especially after inspection of the good vascularity of the urethra during dissection (Figure 3). Several factors may have contributed to poor healing and urinary leakage from the anastomotic site. These include the presence of an aggressive infection with ESBL-producing E. coli, as well as compromised urethral vasculature related to the primary cause of erectile dysfunction and the previous penile prosthesis surgery. In addition, the EPA can further affect urethral vascularity.

To our knowledge, this is the first case report discussing the challenges and risks of urethral surgery in patients with penile prosthesis. In this case, we emphasize urethral healing and the risk of urethroplasty failure in such patients. Careful preoperative planning and intraoperative techniques are crucial to minimize these risks. In some cases, staged procedures or alternative techniques, such as buccal mucosal grafts, may be considered when vascular compromise is a significant concern.^13^

## 4. CONCLUSION

Urethroplasty in patients with a penile prosthesis requires a tailored approach that considers both the urethral stricture and the presence of the implant. In the present case, the patient underwent EPA for a bulbar urethral stricture in the setting of a functioning penile prosthesis. Although early postoperative complications occurred, including urinary leakage and infection with ESBL-producing E. coli, these were effectively managed with prolonged catheterization, suprapubic diversion, and targeted antibiotics. Ultimately, the patient achieved satisfactory urinary flow, and prosthesis function was preserved. This highlights the importance of careful planning and flexible postoperative care.

## COMPETING INTERESTS

The authors have no conflicts of interest to declare.

## AUTHOR CONTRIBUTIONS

All authors made substantial contributions to the conception of the case report, manuscript drafting, and critical revision of the article.
